# Inherited cancer syndromes in adult oncology: a referral guide based on malignant tumor and polyp features

**DOI:** 10.1007/s12094-025-04134-2

**Published:** 2026-02-11

**Authors:** Audrey Guilmot, Magali Belpaire, Eric Olinger, Anne De Leener, Kevin Punie, Francois P. Duhoux

**Affiliations:** 1 Center for Human Genetics, Cliniques universitaires Saint-Luc, Brussels, Belgium; 2 GZA Ziekenhuizen Campus Sint-Augustinus, Antwerp, Belgium; 3 Breast Clinic, King Albert II Cancer Institute, Cliniques universitaires Saint-Luc, Brussels, Belgium; 4 Center for Human Genetics, Centre hospitalier universitaire vaudois (CHUV), Lausanne, Switzerland

**Keywords:** Cancer predisposition, Hereditary cancer syndrome, Genetic counseling, Polyposis syndromes

## Abstract

**Background:**

Due to the challenges of identifying patients with hereditary cancer predispositions in oncology practice, we propose a comprehensive and practical tool intended for oncologists managing adult patients with malignant solid tumors and/or gastrointestinal polyps.

**Methods:**

This tool was developed based on a comprehensive review of international guidelines, GeneReviews, and recent literature. Key criteria for genetic referral were compiled and organized by organ site and tumor subtype.

**Results:**

The resulting resource includes a primary reference table for genetic referral, a polyposis-specific table, two visual summary figures and curated lists of associated genes and syndromes to streamline the clinical decision-making process.

**Conclusions:**

This comprehensive tool aims to support oncologists in the timely identification of patients eligible for genetic counseling, thereby enhancing therapeutic decisions and improving familial risk assessment.

## Introduction

Cancer represents a complex and heterogeneous group of diseases, with its prevalence influenced by multiple factors including age, lifestyle, genetic predisposition, environmental exposures, and healthcare access. Increasingly, germline DNA variations are being recognized as key contributors to the development of malignant solid tumors [1]. Genetic cancer predisposition refers to an increased cancer risk associated with inherited germline pathogenic variants in specific genes.

Although hereditary cancer predispositions represent only a fraction of adult malignancies, monogenic causes constitute a significant proportion in certain tumor types, for example, they are identified in more than 3% of adrenocortical carcinomas and colorectal cancers [2], 5–10% of breast cancers [3], and can exceed 40% in pheochromocytomas/paragangliomas [4].

The primary categories of genes involved in cancer development include oncogenes, tumor suppressor genes, and DNA repair genes [4]. Cancer typically develops through the accumulation of somatic mutations. In contrast, germline mutations are DNA changes present in the egg or sperm cells and affect every cell in the body. These variants typically impact one copy of a tumor suppressor gene from birth, leaving the other copy intact. Inheriting a germline pathogenic variant alone is usually insufficient to trigger cancer. A somatic mutation in the relevant tissues is required to inactivate the second copy of the tumor suppressor gene. This process, described by the Knudson theory, explains why individuals carrying a germline pathogenic variant in a tumor suppressor gene are at a higher risk of cancer compared to the general population [5].

Pathogenic variants in predisposition genes may be inherited from one or both parents and significantly increase cancer risk. As a result, genetic testing and counseling for patients and their families are essential for developing an appropriate and tailored follow-up plan [6]. Predisposition testing can empower individuals to make informed decisions about clinical management, such as enhanced surveillance, risk-reducing surgeries, or targeted therapies. Additionally, this information can be valuable for family members, helping them assess their own cancer risk and take appropriate actions [7].

With the continuous expansion of gene panels and evolving guidelines, oncologists face increasing challenges in deciding which patients should be referred for genetic consultation. This article introduces a practical tool tailored for daily use in adult oncology, focused exclusively on solid tumors and polyp-related syndromes. Designed as a visual and tabular resource, it facilitates rapid assessment of whether a given cancer type or polyp presentation warrants further genetic evaluation.

## Methods

Data are based primarily on the National Comprehensive Cancer Network (NCCN) guidelines, including both “recommended” and “considered” criteria across multiple tumor types [8]. When no formal guideline was available, GeneReviews entries [9] and relevant peer-reviewed publications (e.g., Hampel et al. [10], Benusiglio et al. [11]) were used. All references used to support criteria for referring to a genetic consultation are explicitly listed in the “Guidelines–Reference” column of the main table and for the polyp features (Tables 1 and 6), allowing full traceability and transparency of the criteria applied. To ensure harmonization, when testing criteria overlap across sources, we adopt the broadest clinically useful trigger. For the first figure, we retained the hereditary cancer syndromes for each adult malignant tumor entity listed in Table 1. For the second figure, we associated benign features or tumor for the hereditary cancer syndromes described in the main table based on GeneReviews and cross-checked in OMIM.

## Results

The main table (Table 1) compiles adult (age > 18 years) malignant solid tumors. It is organized alphabetically by anatomical site (organ/system), with subcategorization by specific tumor types, with the exception of sarcomas and gastrointestinal stromal tumors (GIST), which are treated separately due to their cross-anatomical nature. Each row in the table corresponds to an individual tumor type or a clinical situation in which genetic testing should be considered, based on published guidelines or expert consensus, and refers to the associated hereditary cancer syndromes. Importantly, when no specific age threshold is mentioned in the source material, this implies that genetic testing is considered appropriate regardless of age at diagnosis. For clarity, we did not systematically annotate these cases with “at any age,” though that is the intended interpretation. Overlapping indications were resolved by selecting the broadest or most inclusive criteria. As an example, exocrine pancreatic cancer is included regardless of age or family history, as several guidelines now recommend universal genetic testing for this tumor type. This results in the inclusion of multiple syndromes in the same row, each of which may justify genetic referral even in the presence of traditional risk factors.

The benign conditions were excluded (e.g., tuberous sclerosis-associated tumors, pituitary adenomas) except for gastrointestinal polyps, which are addressed in a dedicated table. Pediatric cancer predisposition syndromes or tumor predisposition syndromes with highly penetrant and specific early manifestations were excluded from this adult oncology-oriented tool, as they are typically diagnosed in childhood. This includes, for example, constitutional mismatch repair deficiency (CMMRD), *DICER1* syndrome, and rhabdoid tumor predisposition syndrome type 1 (RTPS1, *SMARCB1*), which usually present with aggressive pediatric tumors such as brain tumors, cystic nephroma, or pleuropulmonary blastoma (DICER1) [12] and atypical teratoid/rhabdoid tumors (RTPS1). In contrast, rhabdoid tumor predisposition syndrome type 2 (RTPS2, *SMARCA4*) was retained, as ovarian small cell carcinoma, hypercalcemic type (SCCOHT), can occur in adolescence or adulthood without prior signs. Although some *DICER1*-associated tumors like Sertoli–Leydig or thyroid carcinomas may arise later, they often follow a diagnosis already made in childhood due to earlier features [11]. Given its variable expressivity and broad pediatric phenotype, *DICER1* was excluded to preserve clinical specificity in this adult-focused reference [13].

**Table 1:** Malignant tumors in adults and genetic syndromes Overview of adult malignant solid tumors associated with hereditary cancer syndromes and genetic referral criteria. Tumors are primarily grouped by organ system and subtype. Gastrointestinal stromal tumors (GISTs) and sarcomas, which are not confined to a single anatomical site, are presented separately. The table is structured in alphabetical order

Cancers	Which tumor types warrant genetic referral and when?	Syndrome or associated cancer predisposition	Guidelines– reference
Adrenal cancer	Adrenocortical carcinoma	MEN1 *Synonym: Wermer syndrome* + Li–Fraumeni syndrome + Lynch syndrome *Synonym*: *hereditary non-polyposis colon cancer*	NCCN [14–16]
Brain/central nervous system Cancer (CNSC)	Glioblastoma diagnosed < age 50	Lynch syndrome *Synonym*: *hereditary non-polyposis colon cancer*	NCCN [16]
	CNSC and synchronous or metachronous Lynch syndrome-related cancer (Table 2)		
	CNSC and 1 FDR or SDR with Lynch syndrome-related cancer (Table 2) diagnosed < age 50		
	CNSC and ≥ 2 FDR or SDR with Lynch syndrome-related cancer (Table 2)		
	CNSC with MMR deficiency determined by PCR, next-generation sequencing, or immunohistochemistry diagnosis		
	CNCS and one additional Li–Fraumeni syndrome tumor (Table 3) in the same person, the first of which occurred < age 46	Li–Fraumeni syndrome	NCCN [14]
	< Age 46 and at least one FDR or SDR with a Li–Fraumeni tumor (Table 3) < age 56		
	Choroid plexus carcinoma		
	Medulloblastoma and ≥ 10 cumulative adenomatous colon polyps in the same person	Familial adenomatous polyposis	Article: Hampel et al. ACMG, 2015 [10]
	Mix of astrocytoma, invasive melanoma, and/or pancreatic cancer in an individual or family	Hereditary melanoma	NCCN [17]
Breast cancer (BC)	≤ Age 50	Hereditary breast and ovarian cancer syndrome	NCCN [14]
	Triple-negative breast cancer		
	Multiple primary breast cancers (synchronous or metachronous)		
	Patients with high-risk early stage or advanced HER2-negative breast cancer who might be candidates for PARP inhibitor		
	In systemic treatment decisions using PARP inhibitors for breast cancer in the metastatic setting		
	Individual of ethnicity associated with higher frequency of specific mutations (e.g., Ashkenazi Jewish)		
	BC and ≥ 3, diagnosis of breast and/or prostate cancer (any grade) on the same side of the family including the patient with breast cancer		
	BC and ≥ 1, close blood relative with any: breast cancer at age ≤ 50; male breast cancer; ovarian cancer; pancreatic cancer; prostate cancer with metastases, or high-, or very-high-risk group		
	Male breast cancer		
	Lobular breast cancer with personal or family history of diffuse gastric cancer	Hereditary diffuse gastric cancer *Synonym*: *diffuse gastric and lobular breast cancer syndrome*	NCCN [14]
	BC and macrocephaly (megalocephaly) (i.e., ≥ 97%, 58 cm in adult female, 60 cm in adult male)	Cowden syndrome *Synonym:* PTEN *hamartoma tumor syndrome*	NCCN [14]
	BC and ≥ 2 major Cowden syndrome criteria (Table 4) in the same person		
	BC and ≥ 3 minor Cowden syndrome criteria (Table 4) in the same person		
	BC and one additional Li–Fraumeni tumor (Table 3) in the same person, the first of which occurred < age 46 (other than breast cancer if the proband has breast cancer)	Li–Fraumeni syndrome	NCCN [14]
	< Age 46 and at least one FDR or SDR with a Li–Fraumeni tumor (Table 3) < age 56		
	< age 31		
	BC and ≥ 1 Peutz–Jeghers polyp in the same person	Peutz–Jeghers syndrome	Article: Hampel et al. ACMG, 2015 [10]
Biliary tract cancer (BTC)	< age 50	Lynch syndrome	NCCN [16]
	BTC and a synchronous or metachronous Lynch syndrome-related cancer (Table 2)		
	BTC and one FDR or SDR with Lynch syndrome-related cancer (Table 2) diagnosed < age 50		
	BTC and ≥ 2 FDR or SDR with Lynch syndrome-related cancer (Table 2)		
	With MMR deficiency determined by polymerase chain reaction, next-generation sequencing, or immunohistochemistry diagnosed		
	Cholangiocarcinoma and another *BAP1*-confirmed tumor (Table 5) in the same person or FDR or SDR	*BAP1* tumor predisposition syndrome *Synonyms:* BAP1 *cancer syndrome; cutaneous/ocular melanoma, atypical melanocytic proliferations, and other internal neoplasms*	GeneReviews [18]
Colorectal cancer (CC)	< Age 50	Lynch syndrome + RPS20-associated hereditary nonpolyposis colorectal cancer syndrome	NCCN [16]
	CC and a synchronous or metachronous Lynch syndrome-related cancer (Table 2)		
	CC and one FDR or SDR with Lynch syndrome-related cancer (Table 2) diagnosed < age 50		
	CC and ≥ 2 FDR or SDR with Lynch syndrome-related cancer (Table 2)		
	With MMR deficiency determined by polymerase chain reaction, next-generation sequencing, or immunohistochemistry diagnosed		
	With a PREMM5* score of ≥ 2.5%		
	CC and ≥ 3 minor Cowden syndrome criteria (Table 4) in the same person	Cowden syndrome *Synonym:* PTEN *hamartoma tumor syndrome*	NCCN [14]
	CC and one major and ≥ 2 minor Cowden syndrome criteria (Table 4) in the same person		
Endometrial cancer (EC)	< Age 50	Lynch syndrome	NCCN [16]
	EC and a synchronous or metachronous Lynch syndrome-related cancer (Table 2)		
	EC and one FDR or SDR with Lynch syndrome-related cancer (Table 2) diagnosed < age 50		
	EC and ≥ 2 FDR or SDR with Lynch syndrome-related cancer (Table 2)		
	With MMR deficiency determined by polymerase chain reaction, next-generation sequencing, or immunohistochemistry diagnosed		
	With a PREMM5* score of ≥ 2.5%		
	EC and macrocephaly (megalocephaly) (i.e., ≥ 97%, 58 cm in adult female, 60 cm in adult male)	Cowden syndrome *Synonym:* PTEN *hamartoma tumor syndrome*	NCCN [14]
	EC and ≥ 2 major Cowden syndrome criteria (Table 4) in the same person		
	EC and ≥ 3 minor Cowden syndrome criteria (Table 4) in the same person		
Eye cancer	Uveal melanoma < age 30	Hereditary melanoma + hereditary breast and ovarian cancer syndrome + *BAP1* tumor predisposition syndrome *Synonyms:* BAP1 *cancer syndrome; cutaneous/ocular melanoma, atypical melanocytic proliferations, and other internal neoplasms*	NCCN [14]
	Uveal melanoma and other primary cancers in the same patient		
Gastric cancer (GC)	Diffuse gastric cancer	Hereditary diffuse gastric cancer *Synonym*: *diffuse gastric and lobular breast cancer syndrome*	NCCN [16]
	< Age 50	Lynch syndrome *Synonym*: *hereditary non-polyposis colon cancer*	NCCN [16]
	GC and a synchronous or metachronous Lynch syndrome-related cancer (Table 2)		
	GC and one FDR or SDR with Lynch syndrome-related cancer (Table 2) diagnosed < age 50		
	GC and ≥ 2 FDR or SDR with Lynch syndrome-related cancer (Table 2)		
	With MMR deficiency determined by polymerase chain reaction, next-generation sequencing, or immunohistochemistry diagnosed at any age		
	GC and gastric polyps in the family following an autosomal dominant inheritance pattern	Gastric adenocarcinoma and proximal polyposis of the stomach	NCCN [16]
	Gastric gastrinoma type 2	MEN1 *Synonym: Wermer syndrome*	NCCN [15]
	Gastric foregut carcinoid and primary hyperparathyroidism or duodenal neuroendocrine tumor or pancreatic neuroendocrine tumor or pituitary adenoma in the same person or in the family history		
GIST	≥ 3 primary GISTs in the same person	Familial gastrointestinal stromal tumor	Article: ACMG [10]
	GIST wild type for *KIT* and *PDGFRA*	Familial paraganglioma/pheochromocytoma *Historically: Carney–Stratakis syndrome or Carney triad*	GeneReviews [19]
Mesothelioma	Malignant mesothelioma and another *BAP1*-confirmed tumor (Table 5) in the same person or FDR or SDR	*BAP1* Tumor predisposition syndrome *Synonyms:* BAP1 *cancer syndrome; cutaneous/ocular melanoma, atypical melanocytic proliferations, and other internal neoplasms*	GeneReviews [18]
Lung cancer (*regardless of smoking history*)	< Age 46 and with a Li–Fraumeni tumor (Table 3) in the same person	Li–Fraumeni syndrome	Article: Benusiglio et al. Lung cancer is also a hereditary disease, 2021 [11]
	< Age 46 and at least one FDR or SDR with a Li–Fraumeni tumor (Table 3) or lung adenocarcinoma < age 56		
	Adenocarcinoma age < 50 with a somatic EGFR-activating variant or deletion		
	T790M, V843I, or V834L EGFR somatic pathogenic variant with an allele frequency of 35–60% in a TKI-naïve patient with lung adenocarcinoma	EGFR-associated genetic susceptibility	Article: Benusiglio et al. Lung cancer is also a hereditary disease, 2021 [11]
	Lung foregut carcinoid and primary hyperparathyroidism or duodenal neuroendocrine tumor or pancreatic neuroendocrine tumor or pituitary adenoma in the same person or in the family history	MEN1 *Synonym: Wermer syndrome*	NCCN [15]
Ovarian/fallopian tube/primary peritoneal cancer	Epithelial ovarian cancer (including fallopian tube cancer or peritoneal cancer)	Hereditary breast and ovarian cancer syndrome + Lynch syndrome	NCCN [14, 16]
	Serous tubal intraepithelial carcinoma	Hereditary breast and ovarian cancer syndrome	NCCN [14, 16]
	Ovarian sex cord tumor with annular tubules	Peutz–Jeghers syndrome	Article: Hampel et al. ACMG, 2015 [10]
	Ovarian small cell carcinoma, hypercalcemic type	Rhabdoid tumor predisposition syndrome	GeneReviews [20]
Pancreatic cancer	Exocrine pancreatic cancer	Hereditary breast and ovarian cancer syndrome + familial pancreatic cancer + Peutz–Jeghers syndrome + Lynch syndrome *Synonym*: *hereditary non-polyposis colon cancer* + hereditary melanoma	NCCN [14, 16]
	Pancreatic neuroendocrine tumor	MEN1 *Synonym: Wermer syndrome* + MEN4 *Synonym: CDKN1B-related multiple endocrine neoplasia*	NCCN [15]
Parathyroid cancer	Parathyroid cancer and primary hyperparathyroidism with age of onset < age 45	Parathyroid carcinoma syndrome + hyperparathyroidism-jaw tumor syndrome	GeneReviews [21]
Pheochromocytoma/paraganglioma	All patients	Familial paraganglioma/pheochromocytoma + MEN2 + Von Hippel–Lindau syndrome	NCCN [15]
Prostate cancer (PC)	Age ≤ 55	Familial prostate cancer + hereditary breast and ovarian cancer syndrome	NCCN [14]
	Metastatic (stage IVB) or node-positive (stage IVA) prostate cancer		
	With very high-risk or high-risk disease		
	PC and ancestry: Ashkenazi Jewish		
	PC and ≥ 3 close blood relatives with prostate cancer (any grade) and/or breast cancer on the same side of the family including the patient with prostate cancer		
	PC and ≥ 1 close blood relative with any: breast cancer at age ≤ 50; male breast cancer; ovarian cancer; pancreatic cancer; metastatic, node positive, or (very) high-risk prostate cancer		
	With intermediate-risk prostate cancer with intraductal/cribriform histology		
Renal/urinary cancer (RC)	Renal cell carcinoma age ≤ 46	Von Hippel–Lindau syndrome	NCCN [22]
	Renal cell carcinoma bilateral or multifocal tumors		
	Renal cell carcinoma and ≥ 1 FDR with renal cell carcinoma		
	Renal cell carcinoma multiple or bilateral chromophobe, oncocytic, and/or hybrid renal tumors	Birt–Hogg–Dubé syndrome	NCCN [22]
	Renal cell carcinoma with multifocal papillary histology	Hereditary papillary renal cell carcinoma	NCCN [22]
	Renal cell carcinoma with fumarate hydratase deficiency or other histologic features associated with fumarate hydratase tumor predisposition syndrome	Hereditary leiomyomatosis and renal cell cancer *Synonyms: fumarate hydratase tumor predisposition syndrome, multiple cutaneous and uterine leiomyomatosis, Reed’s syndrome*	NCCN [22]
	Succinate dehydrogenase-deficient histology	Familial paraganglioma/pheochromocytoma	NCCN [22]
	< Age 50	Lynch syndrome *Synonym*: *hereditary non-polyposis colon cancer*	NCCN [16]
	RC and a synchronous or metachronous Lynch syndrome-related cancer (Table 2)		
	RC and one FDR or SDR with Lynch syndrome-related cancer (Table 2) diagnosed < age 50		
	RC and ≥ 2 FDR or SDR with Lynch syndrome-related cancer (Table 2)		
	With MMR deficiency determined by polymerase chain reaction, next-generation sequencing, or immunohistochemistry diagnosed		
	Renal cell carcinoma and ≥ 3 minor Cowden syndrome criteria (Table 4) in the same person	Cowden syndrome *Synonym: PTEN hamartoma tumor syndrome*	NCCN [14]
	Renal cell carcinoma and one major and ≥ 2 minor Cowden syndrome criteria (Table 4) in the same person		
	Renal cell carcinoma and another *BAP1*-confirmed tumor (Table 5) in the same person or FDR or SDR	*BAP1* tumor predisposition syndrome *Synonyms: cancer syndrome; cutaneous/ocular melanoma, atypical melanocytic proliferations, and other internal neoplasms*	GeneReviews [18]
Sarcoma (non-Ewing soft tissue and osteosarcoma)	Sarcoma and one additional Li–Fraumeni syndrome tumor (Table 3) in the same person, the first of which occurred < age 46	Li–Fraumeni syndrome	NCCN [14]
	< Age 46 and at least one FDR or SDR with a Li–Fraumeni tumor (Table 3) < age 56		
	Rhabdomyosarcoma of embryonal anaplastic subtype		
Sebaceous carcinoma (SC)	< Age 50	Lynch syndrome *Synonym*: *hereditary non-polyposis colon cancer*	NCCN [16]
	SC and a synchronous or metachronous Lynch syndrome-related cancer (Table 2)		
	SC and one FDR or SDR with Lynch syndrome-related cancer (Table 2) diagnosed < age 50		
	SC and ≥ 2 FDR or SDR with Lynch syndrome-related cancer (Table 2)		
	With MMR deficiency determined by polymerase chain reaction, next-generation sequencing, or immunohistochemistry diagnosed		
Skin cancer	≥ 3 primary melanomas in the same person	Hereditary melanoma	NCCN [17]
	Mix of astrocytoma, invasive melanoma and/or pancreatic cancer in an individual or in the family history		
	BAP1-inactivated melanocytic tumors and another *BAP1*-confirmed tumor (Table 5) in the same person or FDR or SDR	*BAP1* tumor predisposition syndrome *Synonyms: cancer syndrome; cutaneous/ocular melanoma, atypical melanocytic proliferations, and other internal neoplasms*	GeneReviews [18]
	> 5 cumulative basal cell carcinoma in the same person	Nevoid basal cell carcinoma syndrome *Synonyms: basal cell nevus syndrome, Gorlin syndrome*	GeneReviews [23]
	Basal cell carcinoma diagnosed < age 30		
Small intestine cancer (SIC)	< Aage 50	Lynch syndrome *Synonym*: *hereditary non-polyposis colon cancer*	NCCN [16]
	SIC and a synchronous or metachronous Lynch syndrome-related cancer (Table 2)		
	SIC and one FDR or SDR with Lynch syndrome-related cancer (Table 2) diagnosed < age 50		
	SIC and ≥ 2 FDR or SDR with Lynch syndrome-related cancer (Table 2)		
	With MMR deficiency determined by polymerase chain reaction, next-generation sequencing, or immunohistochemistry diagnosed		
	Duodenal neuroendocrine tumor	MEN1 *Synonym: Wermer syndrome* + MEN4 *Synonym: CDKN1B-related multiple endocrine neoplasia*	NCCN [15]
Testicular cancer	Sertoli cell cancer	Peutz–Jeghers syndrome	Article: Hampel et al. ACMG, 2015 [10]
Thymus cancer	Thymus foregut carcinoid and primary hyperparathyroidism or duodenal neuroendocrine tumor or pancreatic neuroendocrine tumor or pituitary adenoma in the same person or in the family history	MEN1 *Synonym: Wermer syndrome*	NCCN [15]
Thyroid cancer	Medullary thyroid cancer	MEN2 (A and B) + familial medullary thyroid cancer	NCCN [15]
	Follicular thyroid cancer and macrocephaly (megalocephaly) (i.e., ≥ 97%, 58 cm in adult female, 60 cm in adult male) in the same person	Cowden syndrome *Synonym: PTEN hamartoma tumor syndrome*	NCCN [14]
	Follicular thyroid cancer and ≥ 2 major Cowden syndrome criteria (Table 4) in the same person		
	Follicular thyroid cancer and ≥ 3 minor Cowden syndrome criteria (Table 4) in the same person		
	Papillary or follicular variant of papillary thyroid cancer and ≥ 3 minor Cowden syndrome criteria (Table 4) in the same person		
	Papillary or follicular variant of papillary thyroid cancer and 1 major and ≥ 2 minor Cowden syndrome criteria (Table 4) in the same person		
	Papillary thyroid cancer (cribriform-morular variant)	Familial adenomatous polyposis	NCCN [16]
Uterine cancer	Adenoma malignum of the cervix	Peutz–Jeghers syndrome	Article: Hampel et al. ACMG, 2015 [10]

*BC* breast cancer, *BTC* biliary tract cancer, *CC* colorectal cancer, *CNSC* central nervous system cancer, *EC* endometrial cancer, *FDR* first-degree relative, *GIST* gastrointestinal stromal tumor, *MEN* multiple endocrine neoplasia, *MMR* mismatch repair, *MSI* microsatellite instability, *PC* prostate cancer, *RC* renal/urinary cancer, *SC* sebaceous carcinoma, *SDR* second-degree relative, *SIC* small intestine cancer

*The PREMM 5 model is a clinical prediction algorithm that estimates the cumulative probability of an individual carrying a germline pathogenic variant. https://premm.dfci.harvard.edu/

**Table 2:** Tumors associated with Lynch syndrome [16, 24]

Colorectal adenocarcinoma
Endometrial adenocarcinoma
Urothelial carcinoma (ureter and renal collecting ducts)
Gastric cancer
Ovarian cancer
Small bowel cancer
Brain cancer (usually glioblastoma)
Sebaceous adenocarcinoma
Biliary tract cancer
Pancreatic cancer
Sebaceous adenomas
Keratoacanthomas

**Table 3:** Core tumors associated with Li–Fraumeni syndrome [14, 25]

Breast cancer
Soft-tissue sarcoma
Osteosarcoma
Central nervous system tumor
Adrenocortical carcinoma

**Table 4:** Cowden syndrome criteria [14, 26]

Major criteria
Breast cancer
Endometrial cancer (epithelial)
Thyroid cancer (follicular)
Gastrointestinal hamartomas or ganglioneuromas
Lhermitte–Duclos disease (adult)
Macrocephaly (≥ 97th percentile: 58 cm for adult women, 60 cm for adult men)
Macular pigmentation of the glans penis
Multiple mucocutaneous lesions (any of the following): Multiple trichilemmomas (≥ 3, at least 1 proven by biopsy) ≥ 3 palmoplantar keratotic pits and/or acral hyperkeratotic papules Mucocutaneous neuromas (≥ 3) Oral papillomas (particularly on tongue and gingival), multiple (≥ 3) or biopsy proven or dermatologist diagnosed

Minor criteria
Autism spectrum disorder
Colon cancer
Esophageal glycogenic acanthosis (≥ 3)
Lipomas (≥ 3)
Intellectual deficiency (i.e., intelligence quotient ≤ 75)
Renal cell carcinoma
Testicular lipomatosis
Thyroid cancer (papillary or follicular variant of papillary)
Thyroid structural lesions (e.g., adenoma, nodules, goiter)
Single gastrointestinal harmatoma or ganglioneuroma
Vascular anomalies (including multiple intracranial developmental venous anomalies)

**Table 5:** BAP1 tumor predisposition syndrome-associated tumors (*excluding basal cell cancers and/or cutaneous melanomas, given their high frequency in the general population*) [18]

*BAP1*-inactivated melanocytic tumors
Uveal (eye) melanoma
Malignant mesothelioma
Renal cell carcinoma
Meningioma
Cholangiocarcinoma

In addition to the main table (Table 1), several complementary tools were developed to enhance clinical utility and visualization in clinical workflows. A separate table (Table 6) summarizes the main gastrointestinal polyp features that warrant genetic referral, based on NCCN and ACMG criteria article [8, 10]. This table includes adenomatous, hamartomatous, serrated, and mixed polyposis syndromes, and is intended to assist clinicians in determining when genetic referral is warranted for patients presenting with polyps rather than overt malignancies.

**Table 6:** Polyps and genetic syndromes Genetic referral criteria for patients presenting with gastrointestinal polyps. The table outlines the number, histology, and distribution of polyps indicative of a suspected underlying hereditary polyposis syndromes

Polyps	Which features warrant genetic referral and when?	Guidelines–reference	Syndrome or associated cancer predisposition
Colorectal polyposis, adenomatous	≥ 20 colorectal adenomas in an individual	NCCN [16]	Familial adenomatous polyposis + *MUTYH* associated polyposis + Rare genetic causes of multiple adenomatous polyps [27]
Colorectal polyposis, harmatomatous	≥ 2 cumulative histologically proven Peutz–Jeghers polyps in the same person	NCCN [16]	Peutz–Jeghers syndrome
	Any number of Peutz–Jeghers syndrome polyps in an individual who also has characteristic mucocutaneous pigmentation		
	Any number of Peutz–Jeghers polyps and a positive family history of Peutz–Jeghers syndrome		
	≥ 3 gastrointestinal hamartoma or ganglioneuroma and macrocephaly (megalocephaly) (i.e., ≥ 97%, 58 cm in adult female, 60 cm in adult male) in the same person	NCCN [14]	Cowden syndrome *Synonym: PTEN hamartoma tumor syndrome*
	≥ 3 gastrointestinal hamartoma or ganglioneuroma and ≥ 2 major Cowden syndrome criteria (Table 4) in the same person		
	≥ 3 gastrointestinal hamartoma or ganglioneuroma and ≥ 3 minor Cowden syndrome criteria (Table 4) in the same person		
	Diffuse ganglioneuromatosis of the gastrointestinal tract	Article: Hampel et al. ACMG, 2015 [10]	MEN2
	≥ 5 juvenile polyps of the colon	NCCN [16]	Juvenile polyposis syndrome
	Multiple juvenile polyps throughout the gastrointestinal tract		
	Any number of juvenile polyps with a positive family history of juvenile polyposis syndrome		
Colorectal polyposis, mixed	≥ 10 cumulative polyps with > 1 histology in the same person	Article: Hampel et al. ACMG, 2015 [10]	Hereditary mixed polyposis syndrome
Colorectal polyposis serrated	≥ 5 serrated lesions/polyps proximal to the rectum, all being ≥ 5 mm in size, with ≥ 2 being ≥ 10 mm in size	NCCN [16]	Sessile serrated polyposis cancer syndrome
	> 20 serrated lesions/polyps of any size distributed throughout the large bowel, with ≥ 5 being proximal to the rectum		
Gastric polyps	Gastric polyps restricted to the body and fundus	NCCN [16]	Gastric adenocarcinoma and proximal polyposis of the stomach
	> 100 polyps in the proximal stomach or > 30 polyps in a first-degree relative		
	Predominantly fundic gland polyps and some gastric adenomas, some having regions of dysplasia		

**Fig. 1:**
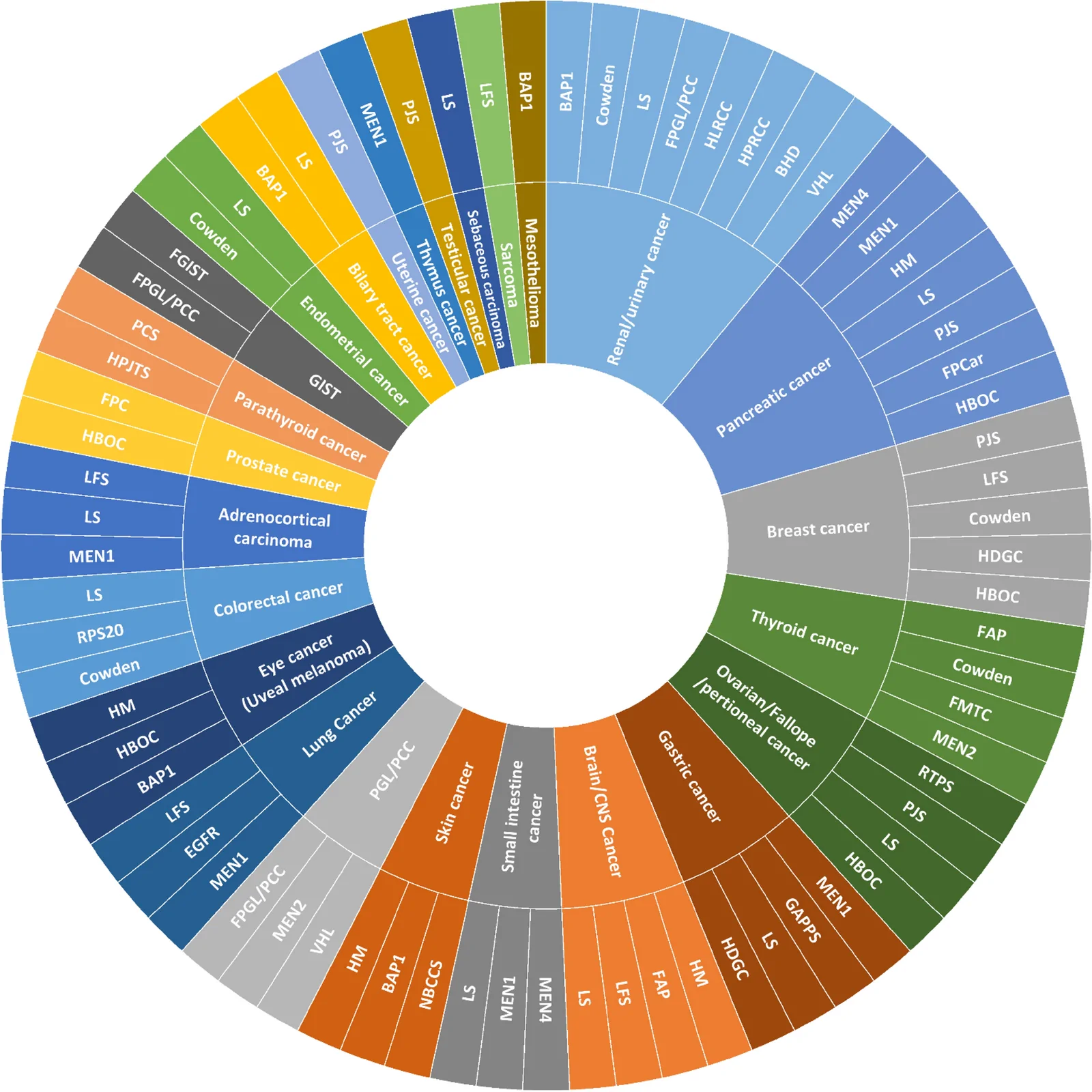
Malignant tumors in adults and genetic syndromes. Visual map of adult malignant solid tumors and their associated hereditary cancer syndromes. *BAP1* *BAP1* tumor predisposition syndrome, *BHD* Birt–Hogg-–Dubé syndrome, *Cowden* Cowden syndrome, *EGFR* EGFR-associated genetic susceptibility, *FAP* familial adenomatous polyposis, *FGIST* familial gastrointestinal stromal tumor, *FMTC* familial medullary thyroid cancer, FPC familial prostatic cancer, *FPCar* familial pancreatic carcinoma, *FPGL/PCC* familial paraganglioma/pheochromocytoma, *GAPPS* gastric adenocarcinoma and proximal polyposis of the stomach, *HBOC* hereditary breast and ovarian cancer syndrome, *HDGC* hereditary diffuse gastric cancer, *HLRCC* hereditary leiomyomatosis and renal cell cancer, *HM* hereditary melanoma, *HPJTS* hyperparathyroidism–jaw tumor syndrome, *HPRCC* hereditary papillary renal cell carcinoma, *LFS* Li–Fraumeni syndrome, *LS* Lynch syndrome, *MEN1* multiple endocrine neoplasia type 1, *MEN2* multiple endocrine neoplasia type 2, *MEN4* multiple endocrine neoplasia type 4, *NBCCS* nevoid basal cell carcinoma syndrome, *PCS* parathyroid carcinoma syndrome, *PJS* Peutz–Jeghers syndrome, *RPS20* RPS20-associated hereditary nonpolyposis colorectal cancer syndrome, *RTPS* rhabdoid tumor predisposition syndrome, *VHL* Von Hippel–Lindau syndrome

Two visual figures are included as practical aids. Figure 1 provides a simplified visual map of malignant solid tumors grouped by anatomical site and their associated hereditary cancer syndromes. It serves as a visual guide or table of contents for Table 1 and uses color coding based on anatomical origin.

**Fig. 2:**
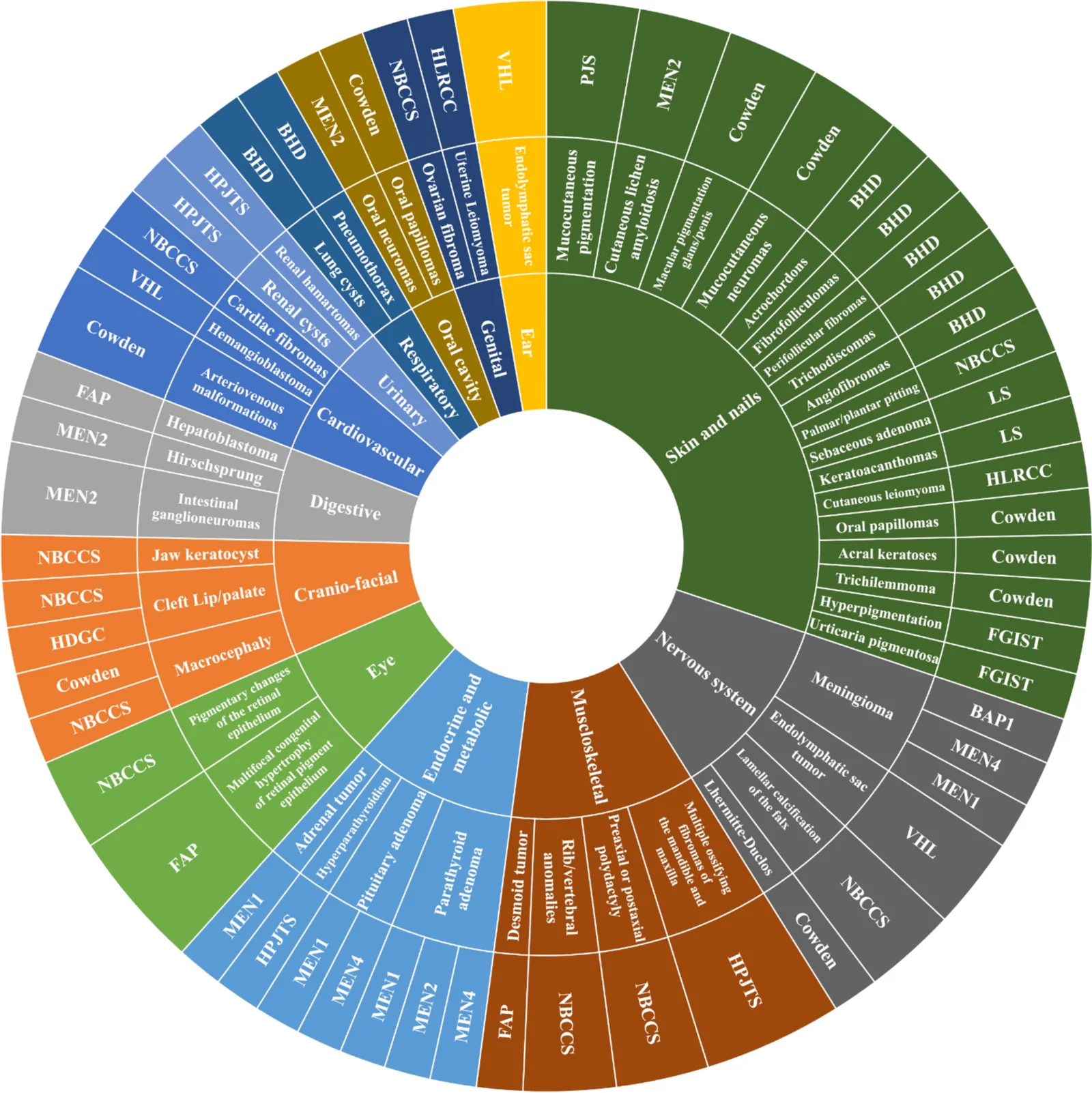
Systemic signs and benign tumors associated with cancer predisposition. Complementary visual tool displaying benign clinical features or findings associated with hereditary cancer syndromes. *BAP1* *BAP1* tumor predisposition syndrome, *BHD* Birt–Hogg–Dubé syndrome, *Cowden* Cowden syndrome, *FAP* familial adenomatous polyposis, *FGIST* familial gastrointestinal stromal Tumor, *HDGC* hereditary diffuse gastric cancer, *HLRCC* hereditary leiomyomatosis and renal cell cancer, *HPJTS* hyperparathyroidism–jaw tumor syndrome, *LFS* Li–Fraumeni syndrome, *LS* Lynch syndrome, *MEN1* multiple endocrine neoplasia type 1, *MEN2* multiple endocrine neoplasia type 2, *MEN4* multiple endocrine neoplasia type 4, *NBCCS* nevoid basal cell carcinoma syndrome, *PJS* Peutz–Jeghers syndrome, *VHL* Von Hippel–Lindau syndrome

Figure 2 illustrates benign clinical features and findings that may be associated with hereditary cancer syndromes. This figure is intended as a complementary reference to help clinicians recognize extra-oncologic signs that could support referral to genetics. However, it does not replace formal criteria and should not be interpreted as a stand-alone indication for genetic testing [18, 21, 23–26, 28–36].

Find hereafter a curated list of genes associated with each hereditary cancer syndrome mentioned in Tables 1 and 6. Each syndrome is linked to one or more genes with established or emerging roles in cancer predisposition based on GeneReviews [9] and OMIM [37]. This list will evolve with advances in scientific knowledge and thus represents the current state of understanding. These genes are not analyzed individually in clinical practice, but rather through massive parallel sequencing gene panel testing (Table 7).

**Table 7:** List of genes associated with each hereditary cancer syndrome mentioned in Tables 1 and 6 [38]

Genetic syndromes	Associated genes
BAP1 tumor predisposition syndrome	*BAP1*
Birt–Hogg–Dubé syndrome	*FLCN*
Cowden syndrome	*PTEN*
EGFR-associated genetic susceptibility	*EGFR*
Familial adenomatous polyposis	*APC*
Familial gastrointestinal stromal tumor	*KIT, PDGFRA*
Familial medullary thyroid cancer	*RET*
Familial pancreatic cancer	*PRSS1*
Familial paraganglioma/pheochromocytoma	*SDHA, SDHAF2, SDHB, SDHC, SDHD*
Familial prostate cancer	*ATM, BRCA1, BRCA2, CHECK2, HOXB13*
Gastric adenocarcinoma and proximal polyposis of the stomach	*APC* (promoter 1B region)
Hereditary breast and ovarian cancer syndrome	*ATM, BARD1, BRCA1, BRCA2, CHEK2, PALB2, RAD51C, RAD51D*
Hereditary diffuse gastric cancer	CDH1, CTNNA1*
Hereditary leiomyomatosis and renal cell cancer	*FH*
Hereditary melanoma	*CDKN2A, CDK4, MITF, POT1*
Hereditary mixed polyposis syndrome	*GREM1* (5’duplication)
Hereditary papillary renal cell carcinoma	*MET*
Hyperparathyroidism–-jaw tumor syndrome	*CDC73*
Juvenile polyposis syndrome	*BMPR1A, ENG, SMAD4*
Li–Fraumeni syndrome	*TP53*
Lynch syndrome	*EPCAM, MLH1, MSH2, MSH6, PMS2*
MEN1	*MEN1*
MEN2	*RET*
MEN4	*CDKN1B*
MUTYH-associated polyposis	*MUTYH*
Nevoid basal cell carcinoma syndrome	*PTCH1, SUFU*
Parathyroid carcinoma syndrome	*CDC73*
Peutz–Jeghers syndrome	*STK11*
Rare genetic causes of multiple adenomatous polyps	*AXIN2, MBD4, MLH3, MSH3, NTHL1, POLD1, POLE*
Rhabdoid tumor predisposition syndrome	*SMARCB1* (RTPS1), *SMARCA4* (RTPS2)
RPS20-associated hereditary nonpolyposis colorectal cancer syndrome	*RPS20*
Serrated polyposis syndrome	*RNF43*
Von Hippel–Lindau syndrome	*VHL*

*CTNNA1 gene has been implicated in some CDH1-negative HDGC families, but with limited penetrance data [39]

## Discussion

This article serves as a clinically grounded framework that aims to clarify when to refer adult oncology patients for consultation in genetics. Together, the tables and figures serve as practical and cohesive tools to help clinicians identify patients who may benefit from genetic referral and testing, thereby supporting more informed and timely decision-making in everyday practice. While this work is based on international guidelines, it is important to note that national recommendations may differ. Such differences should not preclude referral, as the geneticist or genetic counselor will consider these nuances alongside the patient’s full personal and family history. This tool is also designed to evolve, due to its critical use in clinical practice, adapting to advances in genetic research and updates in clinical recommendations. Although this does not fall within the current scope of application, future clinical validation, requiring specific studies, could be considered as the tool evolves. Our goal is to provide a state-of-the-art synthesis that can serve as a foundation for future revisions.

In addition to the clinical criteria outlined in this article, another important indicator stems from tumor genetic analyses. These analyses are increasingly performed to characterize cancers and identify key oncogenic drivers and therapeutic targets. Importantly, some somatic mutations detected through tumor sequencing may suggest an underlying germline pathogenic variant. Their presence should prompt consideration for genetic referral, even in the absence of clinical criteria. This approach, often independent of tumor type, is addressed in complementary frameworks such as the ESMO recommendations by Kuzbari et al. and serves as a valuable adjunct to the tumor-type-based referral strategy presented in this article [40].

When a patient is eligible for germline genetic testing, an increasingly broad set of genes is typically analyzed. To support accurate interpretation and guide panel-based testing strategies, we have included in this article an up-to-date list of genes and their associated syndromes. Today, most hereditary cancer syndromes are evaluated using multigene panels that include a wide range of cancer predisposition genes, regardless of the initial clinical suspicion. While this approach enhances diagnostic yield and efficiency, it also demands careful interpretation. Results must always be considered in the context of the patient’s tumor type and personal or family history. Understanding which gene is associated with which syndrome helps avoid misinterpretation, for example, by preventing the over-reporting of variants of uncertain significance (VUS) in genes unrelated to the patient’s clinical presentation [41]. This distinction is critical to avoid unnecessary anxiety, inappropriate surveillance, or misguided management. Importantly, the list of associated genes is constantly evolving, and what constitutes a strong indication today may shift as new evidence emerges.

Once a monogenic cause is identified in a patient, further follow-up can be initiated in accordance with national and international recommendations. These implications are discussed during the initial genetic consultation and are further detailed when results are communicated. Genetic testing, interpretation of results, and subsequent management must be handled by professionals familiar with genetic counselling, ensuring comprehensive explanation, facilitation of informed consent, and tailored follow-up for the patient. As testing criteria evolve, so do follow-up recommendations. Increasingly, specific genotype–phenotype correlations are being identified, which allows for more personalized clinical management guided by the identified variant. For instance, individuals with *BRCA2* pathogenic variants may receive personalized counseling and surveillance plans depending on the nature and location of the pathogenic variant identified in their family [42].

Beyond follow-up, identifying germline pathogenic variants increasingly guides therapeutic decisions in oncology. Examples include the use of PARP inhibitors in BRCA1/2-associated cancers [43], avoidance of radiotherapy in patients with germline TP53 mutations due to radiosensitivity [44], and consideration of long-term aspirin chemoprevention in Lynch syndrome [45]. Such genotype-driven strategies exemplify the integration of hereditary cancer genetics into precision oncology.

The identification of a monogenic condition may also prompt predictive testing in family members. Such testing helps identify individuals at increased risk of developing cancer, even in the absence of clinical symptoms. This approach is particularly relevant in hereditary cancer syndromes, the vast majority of which follow an autosomal dominant inheritance pattern, conferring a 50% risk of transmission to first-degree relatives. Early diagnosis can enable enhanced surveillance or tailored preventive interventions. When testing of the index patient is not feasible, opening the door to genetic counseling for unaffected first-degree relatives might be recommended. While this is not the primary focus of this article, it is worth mentioning as it provides an additional approach to identifying hereditary risks, even in the absence of a confirmed genetic diagnosis in the index patient. This opens the possibility of better understanding the familial implications of genetic predispositions and providing appropriate surveillance for at-risk family members.

## Conclusion

By providing integrated, visual, and clinically relevant references, this article aims to support oncologists in identifying patients and families who may benefit from genetic assessment. Early identification of hereditary syndromes has important implications for therapeutic decision-making and enables targeted surveillance and prevention strategies for at-risk relatives. It is essential that clinicians remain up to date with evolving genetic guidelines in order to recognize patients—both newly diagnosed and those in remission who remain under their care, who may benefit from referral for genetic consultation and counseling.

## Declarations

### Data availability

Data sharing is not applicable to this article as no new data were created or analyzed in this study.
